# Functional Connectivity of the Pedunculopontine Nucleus and Surrounding Region in Parkinson's Disease

**DOI:** 10.1093/cercor/bhw340

**Published:** 2016-11-22

**Authors:** Ashwani Jha, Vladimir Litvak, Samu Taulu, Wesley Thevathasan, Jonathan A. Hyam, Tom Foltynie, Patricia Limousin, Marko Bogdanovic, Ludvic Zrinzo, Alexander L. Green, Tipu Z. Aziz, Karl Friston, Peter Brown

**Affiliations:** 1 Sobell Department of Motor Neuroscience, UCL Institute of Neurology, Queen Square, London, UK; 2 Nuffield Department of Clinical Neurosciences, University of Oxford, Oxford, UK; 3 Wellcome Trust Centre for Neuroimaging, 12 Queen Square, London, UK; 4 I-LABS MEG Brain Imaging Center, University of Washington, Seattle, WA, USA; 5 Department of Physics, University of Washington, Seattle, WA, USA; 6 Unit of Functional Neurosurgery, UCL Institute of Neurology, Queen Square, London, UK; 7 Nuffield Department of Surgical Sciences, University of Oxford, Oxford, UK; 8 MRC Brain Network Dynamics Unit, University of Oxford, Oxford, UK

**Keywords:** deep brain stimulation (DBS), gait freezing, magnetoencephalography (MEG), coherence, oscillations, pedunculopontine nucleus, human

## Abstract

Deep brain stimulation of the pedunculopontine nucleus and surrounding region (PPNR) is a novel treatment strategy for gait freezing in Parkinson's disease (PD). However, clinical results have been variable, in part because of the paucity of functional information that might help guide selection of the optimal surgical target. In this study, we use simultaneous magnetoencephalography and local field recordings from the PPNR in seven PD patients, to characterize functional connectivity with distant brain areas at rest. The PPNR was preferentially coupled to brainstem and cingulate regions in the alpha frequency (8–12 Hz) band and to the medial motor strip and neighboring areas in the beta (18–33 Hz) band. The distribution of coupling also depended on the vertical distance of the electrode from the pontomesencephalic line: most effects being greatest in the middle PPNR, which may correspond to the caudal pars dissipata of the pedunculopontine nucleus. These observations confirm the crucial position of the PPNR as a functional node between cortical areas such as the cingulate/ medial motor strip and other brainstem nuclei, particularly in the dorsal pons. In particular they suggest a special role for the middle PPNR as this has the greatest functional connectivity with other brain regions.

## Introduction

The pedunculopontine nucleus (PPN), located at the junction between the midbrain and pons, has been studied in a number of species and was first histologically described in humans by Olszewski and Baxter (1982). Rodent studies have shown that it is comprised of cholinergic, glutamatergic, and GABAergic cells (Wang and Morales 2009). Its role in behavior is also heterogeneous—it has been implicated in locomotion in rodents (Garcia-Rill et al. 1987; Skinner et al. 1990) and non-human primates (Nandi et al. 2002), sleep and arousal in cats (Moruzzi and Magoun 1949; Boeve et al. 2007) and humans (Garcia-Rill 1991; Lim et al. 2007, 2009; Arnulf et al. 2010) and reward in rodents (Chen et al. 2006; Norton et al. 2011). Recently, its role as part of the functionally defined mesencephalic locomotor region (an area that generates walking behavior when stimulated in decerebrate animals) has become paramount because of its emergence as a stimulation target in patients with Parkinson's disease (PD) and gait problems (Pahapill 2000; Mazzone et al. 2005; Plaha and Gill 2005; Stefani et al. 2007b; Nandi et al. 2008; Ferraye et al. 2010; Moro et al. 2010; Alam et al. 2011). However, the efficacy of PPN deep brain stimulation (DBS) is variable, and the optimal target location is unclear (Stefani et al. 2007a; Ferraye et al. 2010). One possibility underlying variability in outcome is that different areas in the PPN and surrounding region (PPNR) are functionally coupled with and modulate different brain circuits, only some of which are dysfunctional in human gait disorders (Morita et al. 2014; Li and Zhang 2015). Indeed whether the PPN itself or a neighboring region is “empirically”  the optimal surgical target region remains unclear.

The importance of functional specialization in this region, particularly along the rostro-caudal axis of the PPN, is becoming increasingly apparent in rodent studies (Martinez-Gonzalez et al. 2011; Valencia et al. 2014; Gut and Winn 2015). In humans, functional magnetic resonance imaging (fMRI) and positron emission tomography have insufficient spatial resolution to reliably discern different areas of the PPNR (Ballanger et al. 2009; Snijders et al. 2011). However, direct recordings of local field potentials (LFPs) from intracranial electrodes afford excellent spatial and temporal resolution, and we have demonstrated that the nature of spontaneous oscillatory synchrony picked up in the LFP is topographically organized in the PPNR (Thevathasan et al. 2011, 2012). In particular, we showed that local activity in the alpha frequency band is maximal in a region that lies 2–6 mm below the pontomesencephalic (PM) junction. This area may correspond to the caudal PPN, and specifically to the caudal “pars dissipata”—a diffusely bordered region containing a greater number (but lower concentration) of cholinergic neurons than the central PPN “pars compacta” (Manaye et al. 1999). Importantly, alpha activity in this area correlated with gait performance, and DBS improved gait more when delivered to this area, as opposed to more rostral and caudal regions of the PPNR (Thevathasan et al. 2012). Furthermore, rodent studies have suggested that stimulation of the posterior PPN improves gait, whilst stimulation of the anterior PPN worsens it (Gut and Winn 2015).

Less is known about the functional connectivity of this subregion of the PPN. Studies that have recorded EEG simultaneously with PPNR LFP have been encouraging in so far as functional coupling can be demonstrated between the PPNR and cerebral cortex, but these studies could not associate this coupling with particular cortical and subcortical regions, due to the necessarily limited scalp recording sites in peri-operative patients and the presence of burr holes, which together preclude any spatial mapping (Gaynor et al. 2008; Classen and Schnitzler 2010; Tsang et al. 2010; Thevathasan et al. 2012). Additionally, these studies have not addressed the issue of how the coupling with cortical areas varies within the PPNR.

Here, we characterize how different brain areas couple to the PPNR at rest, taking advantage of recent methodological developments to simultaneously record LFP data from intracranial electrodes and magnetoencephalography (MEG) in a cohort of seven Parkinsonian patients undergoing surgery for PPN DBS. MEG allows dense sampling of brain activity even in post-surgical patients and is resistant to distortion by skull defects, so that cortical localization can be performed in spite of prominent artefacts due to ferromagnetic electrode extension wires (Litvak et al. 2010, 2011a). Accordingly, we test whether PPNR coupling with distant brain areas is frequency-specific, modulated by dopamine and depends upon the position of the electrode within the PPNR. The results are important in understanding the diverse nature of the distributed brain networks involving the PPNR and how these may be organized through spatio-temporal patterning in this region.

## Materials and Methods

### Participants and Surgery

The study was approved by the joint ethics committee of the National Hospital of Neurology and Neurosurgery and the University College London Institute of Neurology and all patients gave their written informed consent. We use a very similar methodology to that previously developed for simultaneous MEG and intracranial recordings from the subthalamic nucleus (Litvak et al. 2010, 2011a). We studied seven (five from Oxford, two from London) patients who had undergone PPN DBS electrode implantation prior to DBS therapy for PD. Six subjects had bilateral implants; although we were not able to record from one electrode in a subject due to a damaged electrode extension wire. One patient had a unilateral implant. Most patients also took part in other experiments which involved electrophysiological recordings—these results have been reported elsewhere (Thevathasan et al. 2012). Clinical details (adapted from Thevathasan et al., 2012) are given in Table 1. Note that EMG recordings performed at the time of MEG demonstrated tremor in subject 4, who was known to have tremor, and in subject 2, who was not known to have clinically significant tremor. However, in both cases, tremor was detectable for less than approximately a third of the time.

**Table 1:** Clinical details of the study participants (adapted from Thevathasan et al. 2011)

Patient	1	2	3	4	5	6	7
Age (years)	55	55	68	70	71	71	69
PD duration (years)	14	25	9	20	20	12	20
UPDRS III (OFF/ON medications)	35/24	33/22	40/26	35/22	37/19	38/20	50/23
IT27-30 (OFF/ON medications)	7/6	6/5	11/8	6/5	10/5	na	8/6
Preoperative/postoperative GFQ	55/38	36/15	49/42	36/28	na	30/na	na
FOGQ	22	15	24	13	na	11	na
FallsQ	4	3	2	3	na	3	na
Levodopa dose equivalent (mg/day)	1600	300	1650	900	1450	1200	1950
Supportive for UK Brain Bank criteria^a^	A,P	D,A,P	A,P	D,A,T,P	D,A,T,P	D,P	D,A,P
PPNR electrodes recorded	R only	L+R	L+R	L+R	R only	L+R	L+R
Clinically chronically used electrodes	R2	L01 R12	L12 R12	L1 R1	na	L23 R0	L1 R1
Rest trials per condition (OFF/ON medications)	45/22	101/53	102/102	99/102	–/105	51/–	94/97
Percentage of total recorded data rejected due to artefacts (%)	7/7	6/3	5/3	3/3	–/2	3/–	9/6

GFQ, Gait and Falls Questionnaire (score/64); FOGQ, Freezing of Gait Questionnaire (score/24); Falls Q, Falls Questionnaire (score/4); NA, not assessed. For all motor scales, higher scores indicate worse function. Key to UK Brain bank criteria; D, dyskinesias; A, asymmetry persistent; T, tremor at rest; P, progressive disease course.

All patients had bradykinesia. All patients were operated in Oxford except patients 5 and 7, who were operated in London. All patients were male. All subjects had bilateral PPNR implants except subject 5, who had a unilateral right PPNR implant. We were only able to record from the right PPNR in subject 1 due to a damaged electrode extension wire. Rostro-caudal stimulation location and clinical outcome data for all but two of these subjects has been reported previously, with cases 1–5 corresponding to cases 1 and 3–6 in Thevathasan et al. (2011). Outcome was assessed by a drop in GFQ postoperatively. Outcome in patient 5 was assessed with UPDRS II items scoring freezing, falls and gait with the combined score being 5/16 preoperatively and 4/16 postoperatively (ON medication). UPDRS III = part III (motor) of the Unified Parkinson's disease rating scale (score/108). IT27-30 = items 27–30 UPDRS III assessing gait, posture, and balance (score/16). The electrode contacts that were used clinically are also presented in the format of electrode side, followed by contact number(s), Contacts are numbered according to the convention “0” for the most caudal contact, followed by “1”, “2” and then “3” in rostrally ascending order. For example, “R01” refers to bipolar stimulation of the deepest and second deepest contact on the right, whilst “L3” refers to monopolar stimulation of the most rostral contact on the left. All participants perceived enough symptomatic benefit to continue stimulation except subject 4 where stimulation was discontinued. We also include the number of rest trials per condition and the percentage of data removed per session due to artefact.

^a^Additional to disease duration and levodopa response as documented elsewhere in the table.

The indications for surgery were PD with predominant levodopa-unresponsive gait impairment and/or falls due to either freezing or postural instability. Gait freezing and postural instability are common features of PD, especially as the disease progresses (Giladi et al. 2001; Bloem et al. 2004). However, medication-resistant gait freezing may also be due to atypical pathologies (Hallett 2008; Jankovic 2008). In the absence of a definitive test in life, we stress that the diagnosis of PD in our cohort is presumptive.

Prior to surgery, the motor impairments of all patients were evaluated using part III of the Unified Parkinson's disease Rating Scale (UPDRS) after omitting all dopaminergic medication overnight and then following administration of 200 mg of levodopa. Patients treated in Oxford also prospectively completed the Gait and Falls Questionnaire (GFQ, score/64) which assesses Parkinsonian gait disturbance including gait freezing, festination, and falls. The Freezing of Gait Questionnaire (FOGQ, score/24) and Falls Question (FallsQ, score/4) are components of GFQ (Giladi et al. 2000, 2009). The London patients were assessed with UPDRS (part II) items assessing gait, freezing and falls (combined score/16). For all motor scales, higher scores indicate worse function.

**Figure 1.**
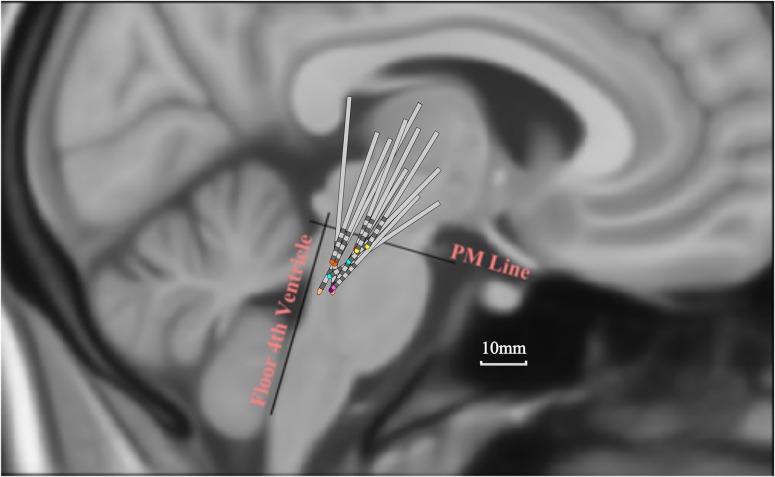
DBS electrode contact locations within the brainstem. Locations are represented in MNI space (sagittal view). PM, Pontomesencephalic line connecting the PM junction to the caudal end of the inferior colliculi. Electrodes from different subjects have different colored tips. Not all contacts are within the PPN, affording us the opportunity to divide the sampled brainstem region according to height with respect to the PM line. Note that this figure is adapted from Thevathasan et al. (2011). Flair MRI of case 2 showing axial slices at different depths is illustrated in Supplementary material to Thevathasan et al. (2011).

Techniques to target and implant DBS electrodes in the PPN have been described previously (Pereira et al. 2008; Zrinzo et al. 2008; Foltynie and Hariz 2010). In this study, preoperative stereotactic imaging (stereotactic proton density weighted magnetic resonance imaging (MRI) in London, and stereotactic computerized tomography (CT) fused with T2 weighted MRI in Oxford) were used to target the PPN medial to the lemniscal system and lateral to the superior cerebellar peduncle and its decussation. The DBS electrode used was model 3389 (Medtronic, Minneapolis, MN) with four platinum–iridium cylindrical contacts of diameter 1.27mm, length 1.5 mm, and centre-to-centre separation 2 mm. After implantation, electrodes were connected to an accessory kit, typically both connectors being tunneled to the left temporoparietal area and externalized through the frontal region. The percutaneous extension wires were made of stainless steel and generated artefacts in the MEG signals as previously reported (Litvak et al. 2010). Although non-magnetic extension wires also exist and have been used in several studies by another group (Hirschmann et al. 2011, 2013a, 2013b), these were not available to us for the present study due to licensing issues in the UK. No microelectrode recordings were made. To confirm correct placement, electrodes were visualized on immediate post-operative imaging with the surgical frame in situ (proton density weighted MRI in London and CT (1mm slice thickness) fused with preoperative T2 weighted MRI in Oxford). To facilitate comparison across subjects, the postoperative images were linearly transformed into Montreal Neurological Institute (MNI) space using the fMRIB Software Library (Smith et al. 2004). The coordinates of each contact were determined (in millimetres) relative to the midline (*x*, laterality), ventrodorsal distance from floor of the fourth ventricle (*d*, anteroposterior location) and rostro-caudal distance from a PM line connecting the PM junction to the caudal end of the inferior colliculi (*h*, height), as described previously (Ferraye et al. 2010). Lead locations are summarized in Fig. 1.

### Simultaneous PPNR-LFP and MEG Recordings

MEG recordings were performed in London with the 275 channel CTF (VSM MedTech Ltd.) or in Oxford with the 306 channel Elekta (Elekta Oy) systems. Simultaneous to the MEG recording, both right and left first dorsal interosseus electromyographic (EMG) signals, and four intracranial LFP channels were recorded per electrode. Different amplifiers were required to acquire LFP data from subjects undergoing MEG scanning in different machines. However, offline analysis (see below) was designed to make the data directly comparable. EMG and LFP recordings from the first London patient recruited were referenced to the right mastoid and acquired using the integrated EEG system. Similar data from the second London patient were acquired using a Brainamp MR system (Brain Products GmbH) and merged with the MEG data offline using a common synchronization signal (white noise) recorded on both systems (Oswal et al. 2016b). In Oxford, LFP signals in the PPNR were acquired in a bipolar configuration via a Digitimer D360 amplifier (Digitimer Ltd.) connected to the MEG auxiliary input channels, and high-pass filtered at 0.5 Hz. All data were sampled at 2400 Hz and stored to disk for subsequent offline analysis. MEG, LFP, and EMG data were hardware high-pass filtered at 0.03 Hz (Oxford MEG and EMG) or 1 Hz (London EMG and LFP; Oxford LFP only) and low-pass filtered at 600 Hz (all signals). London LFP recordings were converted offline to a bipolar montage between adjacent contacts (three bipolar channels per side) to limit the effects of volume conduction from distant sources (Oxford LFP recordings were already recorded in this format).

Recordings were performed between 2 and 6 days post-operatively, after omitting all dopaminergic medication overnight (OFF condition). The recording was then repeated approximately 30–60 mins after administration of 200 mg levodopa (ON condition). One subject from London only completed the ON recording and one subject from Oxford—only OFF (see Table 1). Each recording comprised rest blocks followed in some of the cases by task blocks. In this report we focus only on the resting data, which were collected in two blocks lasting 3 min each (although some subjects were able to complete only one block in some sessions—see Table 1 for final trial numbers per patient per condition). During the rest block the patients were asked to keep still, relax with their eyes open and focus on a fixation cross. A neurologist was present in the magnetically shielded room during the experiment to monitor the patient's well-being. It is worth noting that due to the often frail nature of these patients and the short inter-operative period, we performed only one recording session, and were therefore unable to counterbalance the order of the ON and OFF conditions.

### Data Conversion and Artefact Detection

The data were analysed using SPM12 (http://www.fil.ion.ucl.ac.uk/spm/, Litvak et al. 2011b), Fieldtrip (http://www.ru.nl/neuroimaging/fieldtrip/, Oostenveld et al. 2011) and Data Analysis in Source Space (http://www.fil.ion.ucl.ac.uk/spm/ext/#DAiSS) toolboxes. The continuous resting recording was converted to SPM format. Artefacts such as flats and jumps were detected in each channel and marked in the continuous data (see Supplementary material). Channels where more than 80% of the data were marked as bad were not used for further analysis (but see below). This step was done prior to any further processing to avoid distortion of artefact patterns by e.g. digital filtering.

### Spatiotemporal Signal Space Separation

Unlike the CTF system which has high dynamic range and can record the artefacts generated by DBS leads with no saturations (Litvak et al. 2010), the Elekta MEG system channels did show clipping. This was more common for sensors overlying the location of the percutaneous extension wires under the skin and more severe for magnetometers due to their greater sensitivity to distant sources and environmental noise. Complete exclusion of all channels or trials containing saturations or jumps would lead to excessive data loss. We, therefore, used Spatiotemporal Signal Space Separation (tSSS, Taulu and Simola 2006) to clean the data of artefacts using as much valid information in the data as possible. We used Matlab implementation of tSSS provided to us under license by Elekta. This was the prototype code for the commercial implementation available in the MaxFilter software. Full details are given in Supplementary Materials.

### Further Preprocessing

From this point onwards the analysis procedures will be slightly different for London (CTF) and Oxford (Elekta) data. The CTF data contained no saturations and few jumps but they could not be subjected to tSSS because this procedure is only implemented for the Elekta system. Thus, these data remained contaminated by artefacts and their analysis was based on our previously established techniques for optimal use of beamforming for artefact suppression (Litvak et al. 2010, 2011a). The Elekta data were rendered artefact-free by tSSS but to subsequently combine the two sensor types and account for the fact that the channel data were rank-deficient we had to use a different approach to beamforming—SSS-BF (Vrba et al. 2010).

Following artefact marking and tSSS for Elekta data or artefact marking alone for CTF data the continuous data were high-pass filtered above 1 Hz and the line noise artefacts at 50 Hz and all harmonics up to 550 Hz were removed using notch filters (fifth order zero-phase Butterworth filters with 4 Hz notch bandwith). The data were then divided into arbitrary 3.4 s trials. The choice of the trial length was for consistency with our previous study (Litvak et al. 2011a) where 3.4 s equalled 1024 samples. Although in the present study this was no longer the case, this trial length seemed to work well.

Following epoching, trials containing artefacts in the PPNR-LFP data were excluded from further analysis. These artefacts were detected by thresholding the absolute LFP amplitude. The threshold was typically 30 uV but could be adjusted for individual subjects based on examination of the data. Additionally, for CTF data, trials where any jumps were marked previously in the MEG data were also excluded. Final trial numbers following artefact removal are presented in Table 1.

### Sensor-level Analysis

Coherence was the principal measure of functional connectivity used in this study. It provides a frequency-domain measure of the linear phase and amplitude relationships between signals (Thatcher et al. 1986; Rappelsberger and Petsche 1988; Shen et al. 1999; Buzsaki and Draguhn 2004; Magill et al. 2006).

In order to check for existence of coherence between PPNR-LFP and MEG and define frequency bands for subsequent source analysis we performed statistical analysis of coherence for individual subjects and contacts at the sensor level. The procedure was identical to that previously described (Litvak et al. 2011a). Briefly, we computed coherence between each of the bipolar PPNR-LFP channels and MEG channels. For the Elekta data we only used magnetometers which have similar sensitivity profiles to the axial gradiometers of CTF. Note, however, that as part of tSSS the magnetometer data were re-computed based on both magnetometers and planar gradiometers of the original data. Spectral analysis for coherence computation was done using the multi-taper method (Thomson 1982; Mitra and Pesaran 1999). We analysed data from 1 to 45 Hz with frequency resolution of 2.5 Hz. Channel × frequency coherence spectra were converted into volumetric scalp × frequency images for statistical analysis in SPM (Kilner and Friston 2010; Litvak et al. 2011b) and saved in Neuroimaging Informatics Technology Initiative format. The images were smoothed with a Gaussian kernel of 10 mm × 10 mm × 2.5 Hz. For each original image 10 surrogate images were generated by randomly reshuffling the PPNR-LFP data across trials and repeating the above steps.

Two-sample *t*-test with equal variance assumption was performed between the original image and the surrogates and significant differences were detected at the level of *P* < 0.01, one-tailed, FWE corrected at the peak level with extent threshold of 100 voxels.

**Figure 2.**
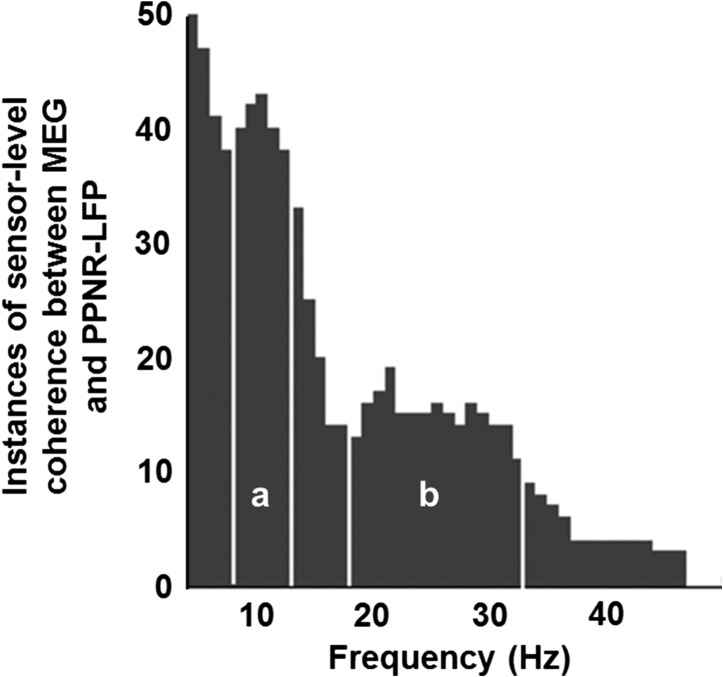
Individual sensor-level analysis. Sensor-level coherence maps were generated by calculating MEG sensor coherence with individual PPNR-LFP subjects and contact pairs. These maps were tested against shuffled surrogate data to identify significant clusters of sensor-level coherence. The frequency ranges of these clusters were used to generate this histogram which shows how often each frequency bin is included in significant clusters of coherence. Two peaks were identified:  (a) alpha coherent sources occurred predominantly between 8 and 12 Hz and (b) beta coherent sources occurred predominantly between 18 and 33 Hz. The boundaries of these ranges are shown on the histogram as white vertical lines. These same frequency ranges were then used to generate SPMs for group analysis.

Although the suprathreshold clusters produced by the statistical analysis had both spatial and frequency dimensions, we initially only used their corresponding frequency bands. These bands were pooled across all subject/contact/drug state combinations and used to generate a histogram showing how often each frequency bin was included in a significant coherence cluster. Based on this histogram (see Fig. 2) we defined the bands for group analysis at the source level. These data were subsequently also used to describe the variability of the nearest individual source locations to each canonical location derived from the group analysis (see Supplementary Material and Fig. S1).

### The Beamformer Approach to Coherent Source Localization

Cortical sources coherent with PPNR-LFP activity were located using the Dynamic Imaging of Coherent Sources (DICS) beamforming method (Gross et al. 2001). Using beamforming, coherence can be calculated between each PPNR-LFP channel and a 3-dimensional grid of points representing potential sources within the brain (Gross et al. 2001). The beamforming method is based on the linear projection of sensor data using a spatial filter computed from the lead field of the source of interest and either the data covariance (time domain) (Van Veen et al. 1997) or cross-spectral density matrix (frequency domain) (Gross et al. 2001). For CTF data original channel data were used as input to DICS and prior to filter computation the cross-spectral density matrix was regularized with regularization coefficient of 0.01% of the mean of the diagonal matrix elements (Litvak et al. 2010). For Elekta data a different approach had to be used due to the different nature of the data which were rank-deficient (following tSSS) and divided across two sensor types. Furthermore, since these data were free of large deflections generated by metal artefacts the regularization parameter optimized for artefact-contaminated data would not be applicable for them. We, therefore, used SSS-BF approach (Vrba et al. 2010). Prior to beamforming the exact same data as used for the sensor-level analysis were projected back into multipole space. For this purpose the previously computed and stored SSS projection matrix was further regularized by iteratively removing the one multipole basis vector, whose removal reduced the condition number (ratio of the largest and smallest eigenvalue) of the projection matrix the most, until the condition number was below a pre-specified threshold (80 in our case). This procedure was suggested in the SSS-BF paper (Vrba et al. 2010) and we found it essential to produce sensible DICS images, although the exact value of the threshold parameter did not have a strong effect on the results. The number of the basis vectors was reduced from original 80 to between 60 and 75 depending on the dataset (mean 70).

Lead fields were computed using a single-shell head model (Nolte et al. 2004) based on an inner skull mesh derived by inverse-normalizing a canonical mesh to the subject's individual preoperative MRI image (Mattout et al. 2007). Co-registration between the MRI and MEG coordinate systems used three fiducial points: nasion, left, and right pre-auricular; see Litvak et al. (2010) for further details. The coherence values were computed on a 3-dimensional grid in MNI space with spacing of 5 mm bounded by the inner skull surface. Global normalization was then performed by dividing the coherence value at each grid point by the mean value computed across the whole grid (Litvak et al. 2011a). This potentially removes confounds related to nuisance variations in signal-to-noise ratio such as variable head distance from MEG coils and the use of different MEG systems. However, it also constrains the analysis to distinguish changes in the relative topography of coherence, rather than absolute values. The resulting normalized values were linearly interpolated to produce volumetric images with 2 mm resolution. These images were further smoothed with an 8 mm isotropic Gaussian kernel.

### Localization of Frequency-specific Areas of Brain Coherence with the PPNR

The aim of this analysis was to determine if the topography of cortico-pedunculopontine region coherence was frequency dependent. To allow group comparison of data, coherence DICS images were generated in all patients over two fixed frequency bands determined based on the sensor-level analysis (see Results): alpha (8–12 Hz), beta (18–33 Hz). Individual whole brain DICS coherence images were created for each of the three bipolar PPNR-LFP channels per side per patient. Left PPNR DICS coherence images were reflected across the median sagittal plane to allow comparison of ipsilateral and contralateral sources to the PPNR regardless of original PPNR side.

We proceeded statistically in two steps—first we identified brain regions with significant coherence with the PPNR and secondly we identified the effect of electrode placement and dopamine on coherence at these peak brain locations.

For each frequency band, we identified brain areas coherent with the whole of the PPNR by comparing genuine DICS images with surrogate images using standard procedures to produce statistical parametric maps (SPMs). For each original DICS image, a surrogate DICS image was generated by randomly reshuffling the PPNR-LFP data across trials and repeating the above steps. For each frequency band (alpha and beta), the coherence data at each voxel of the DICS images were subjected to a fixed-effects ANOVA with image type (genuine coherence vs. surrogate coherence) as a factor of interest and dopamine (ON vs. OFF), PPNR electrode location (see below), subject and PPNR side as nuisance factors. Post hoc one way *t* contrasts of genuine—surrogate images thresholded at *P* < 0.05 FWE and cluster size above 150 voxels were used to identify brain regions with significant coherence with the PPNR. Although, at this stage we were interested in only the genuine versus surrogate comparison, as a precaution we included other factors in the model so as not to bias the peak locations identified towards any particular electrode location or dopamine state (a potential problem with unbalanced designs).

### Grouping of Bipolar Contact Height within the PPNR

We grouped the location of electrodes within the PPNR according to the following strategy. PPNR electrode location was most variable in the rostro-caudal plane which we have termed the height *h* of the contact (i.e., the distance from the PM line, Ferraye et al. 2010). In our cohort, the height of contact placement, varied between −12.4 and +5.0 mm relative to the PM line (see results) and so we focused on this plane only for further analysis. Given our DICS images were derived from “bipolar” PPNR-LFP recordings, we took the mean height of both electrode contacts to be the height of the recorded LFP. Previous PPNR-LFP recordings suggest that centrally placed bipolar contacts, between −2 and −6 mm relative to the PM line, exhibit greater local alpha power and that activity in this region correlates with gait speed (Thevathasan et al. 2012). Therefore, we were particularly interested in coupling with this, potentially clinically important, subregion within the PPNR. To investigate this we grouped bipolar contacts into three categories depending on whether their height: above −2 mm, termed the upper PPNR (including the rostral PPN); −2 to −6 mm, termed the middle PPNR (including the caudal PPN); and below −6 mm termed the lower PPNR (including the region below the caudal PPN). We note that we use the term PPN and surrounding region (PPNR) simply to describe the distribution of electrode locations in our clinical cohort, and that it does not connote a particular anatomical region in of itself.

### Source Data Analysis

Virtual electrode time series were extracted from the individual power peaks (termed the “source”) closest to the group coherence peak for each band using LCMV beamformer. The beamforming procedures were as described above for DICS except the covariance matrix rather than band-limited cross-spectral density matrix was used for filter computation. We then computed coherence between PPNR-LFP and the virtual electrode time series to confirm the existence of peaks consistent with DICS results. The resulting coherence values were then subjected to a mixed-effects ANOVA with subject as a random factor, and dopamine (ON vs. OFF) and electrode height (above −2 mm, between −2 and −6 mm, below −6 mm) as fixed factors. Each frequency band (alpha and beta) was modeled individually and main effects and the interactions were tested using SPSS (Version 22, IBM Corp) with a threshold of *P* < 0.05. Post hoc *t-*tests were used to investigate significant effects and were thresholded at *P* < 0.05 equivalent using a Bonferroni correction (SPSS determines this by multiplying the uncorrected *P-*value by the number of contrasts, rather than dividing the alpha by the number of contrasts).

We also addressed the question whether there was a delay between PPNR-LFP and virtual electrode activity indicative of an effect of true neuronal connectivity rather than volume conduction of electrical signals. For this purpose we computed the imaginary part of the coherency (Nolte et al. 2004).

## Results

### Intracranial Electrode Recordings Correspond to Activity of the PPN and Neighboring Regions

Electrode positions are summarized in Fig. 1. The mean ± standard deviation (std) distance from the PM line (*h*, height) was −4.7 ± 4.5 mm, the mean anteroposterior distance (*d*) was 6.3 ± 2.2 mm and the mean laterality (*x*) was −6.2 ± 1.5 mm on the left side and 8.9 ± 2.5 mm on the right side. The greatest anatomical variation in contact location (i.e., the greatest std) was, therefore, along the rostrocaudal (*h*) axis. This variability along the rostrocaudal axis means that many electrode contacts lay outside the assumed boundaries of the PPN, affording us the opportunity to examine the distribution of subcortico-cortical coherence with respect to this broad area, which presumably includes surrounding structures and which we have termed the PPN and surrounding region (PPNR). We thus divided this region covered by electrode contacts into three subregions and considered the nature of subcortico-cortical coherence with respect to these. The three subregions were the 1) upper PPNR (e.g., structures near to and including the rostral PPN); 2) middle PPNR (e.g., structures near to and including the caudal PPN) and 3) lower PPNR (e.g., structures caudal to the PPN proper).

Still the above detail would only be useful if LFPs were focal, allowing discrimination between subregions. Both alpha and beta band power were relatively focal. Alpha power dropped to 46.3 ± 6.7 (SEM)% from the contact with peak alpha power to the mean of the remaining contacts on each electrode. Beta power similarly dropped to 48.3 ± 6.4%.

### Networks Involving the PPNR Are Frequency Specific

In order to check for existence of coherence between PPNR-LFP and MEG and define frequency bands for subsequent source analysis we performed statistical analysis of coherence for individual subjects and contacts at the sensor level. Coherence between the MEG sensors and PPNR-LFP was calculated and the resulting coherence images (2-dimensional scalp × frequency images) were compared with 10 surrogate images using a *t-*test thresholded at *P* < 0.01 FWE using SPM software. The frequency ranges of significant clusters of coherence were pooled across subject/contact/drug state, and a histogram was generated that showed how often each frequency bin was included in a significant coherence cluster. The results are presented in Fig. 2. Inspection of the data suggests that coherence is focused in two main frequency bands—the alpha band at 8–12 Hz and the beta band at 18–33 Hz. These two frequency ranges were taken forward into the subsequent analysis. We did not include theta coherence because this range may be heavily contaminated with ferromagnetic artefact from DBS extension wires.

### Brain Areas Showing Frequency-specific Coherence with the PPNR

**Figure 3.**
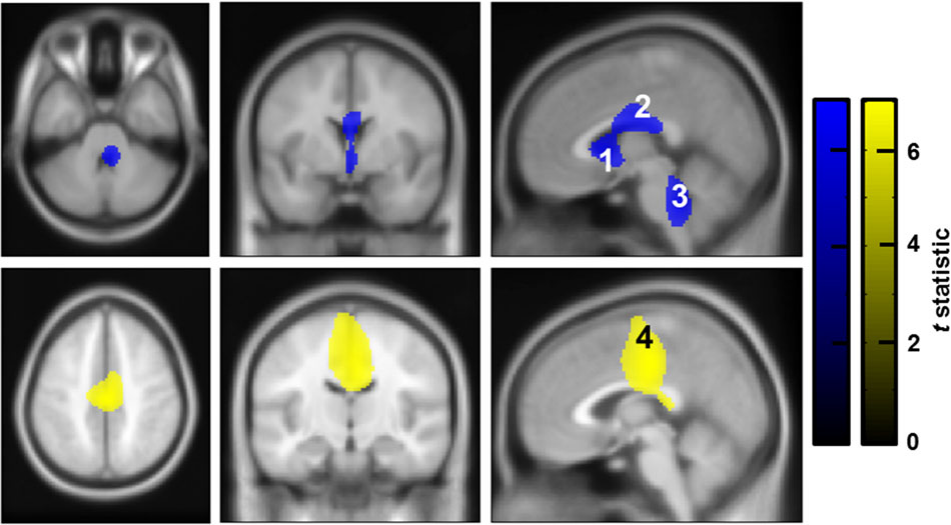
Brain regions with significant coherence with the PPNR. Frequency-specific whole brain images of coherence with the PPNR (DICS images) were entered into an ANOVA with surrogate shuffled data. Note that all images from left-sided PPNR-LFP data have been reflected across the median sagittal plane to allow inference regarding ipsilateral versus contralateral areas of coherence. The resulting thresholded SPMs are displayed above, overlayed onto corresponding orthogonal sections through an averaged T1 weighted MRI in MNI space. Each significant cluster has been labeled with a corresponding number, which corresponds to the ROI label. Top row (blue): alpha coherence with the PPNR was greater than surrogate coherence in the subgenual cingulate (ROI 1), the posterior midcingulate (ROI 2) and the posterior brainstem (ROI 3). Bottom row (yellow): Beta coherence with the PPNR was significantly greater than surrogate coherence in the medial frontal wall in an area that overlapped with the posterior midcingulate, the SMA and the leg area of the primary motor cortex (ROI 4). Corresponding peak location statistical data are presented in Table 2. The color bar represents the *t* statistic for alpha (blue) and beta (yellow) coherence. Images are thresholded to *P* < 0.05 (FWE corrected).

After determining the “frequency ranges” of significant coherence between the PPNR and the rest of the brain, we proceeded to localize the brain *areas* involved. We used the standardized frequency ranges from the previous step (alpha [8–12 Hz] and beta [18–33 Hz]) to generate DICS images of whole brain—PPNR coherence for each contact and frequency band. These were compared with surrogate DICS images within an ANOVA framework (one per frequency band) using standard SPM methods thresholded at *P* < 0.05 FWE (see Fig. 3). Alpha coherence with the PPNR was greater than surrogate coherence in the subgenual cingulate (labeled Region of Interest 1 [ROI 1]), the posterior midcingulate (ROI 2) and the posterior brainstem mostly in the dorsal pons (ROI 3) (see Fig. 3 and Table 2). Beta coherence with the PPNR was significantly greater than surrogate coherence in the medial frontal wall in an area that overlapped with the posterior midcingulate, the supplementary motor area (SMA) and the leg area of the primary motor cortex (ROI 4) (see Fig. 3 and Table 2). Statistical data for the peak voxel in each ROI are presented in Table 2.

**Table 2:** Peak voxel co-ordinates and statistics from the SPM analysis

Frequency	Label	Location	Peak co-ordinates	Statistic	*P-*value
Alpha	ROI 1	Subgenual cingulate	0, 10, 2	*t*(1,103) = 5.51	<0.001
	ROI 2	Posterior midcingulate	2, −6, 24	*t*(1,103) = 5.03	0.005
	ROI 3	Brainstem (maximal in dorsal pons)	6, −40, −36	*t*(1,103) = 5.31	0.002
Beta	ROI 4	Motor strip and neighbouring areas	0, −20, 44	*t*(1,103) = 6.47	<0.001

SPMs were generated showing brain voxels where coherence was greater than surrogate shuffled data. Separate SPMs were generated for alpha and beta frequency coherence. The analysis was thresholded at *P* < 0.05 FWE and peak voxels in the four surviving clusters (labeled as ROIs) are shown above. ROIs 1–3 show significant alpha coherence, whereas ROI4 shows significant beta coherence with the PPNR.

### Coherence is Topographically Modulated within the PPNR

**Figure 4.**
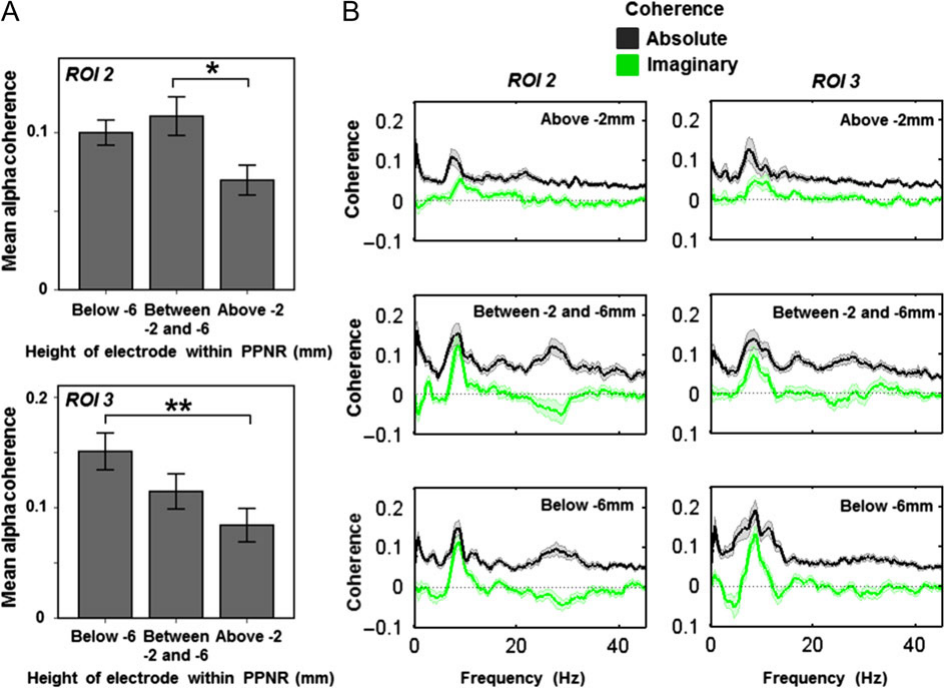
Alpha coherence varies within the PPNR. Source activity was extracted from the peak voxel of alpha coherence, taken from ROI 1 (subgenual cingulate), ROI 2 (posterior midcingulate) and ROI 3 (brainstem, maximal in dorsal pons). For each ROI, mean alpha coherence was then calculated and the resulting values were subjected to a mixed-effects ANOVA, with subject as a random factor, and dopamine (ON vs. OFF) and electrode height as factors of interest. Electrode height within the PPNR was split into three groups (above −2 mm termed the upper PPNR (including the rostral PPN); −2 to −6 mm termed the middle PPNR (including the caudal PPN); and below −6 mm termed the lower PPNR (including the region below the caudal PPN). The main effect of height was significant for PPNR coherence with ROIs 2 (*A* Top) and 3 (*A* Bottom) but not for ROI 1 (data not shown). *A* Top: post hoc *t-*tests showed that coherence with ROI 2 was higher in the middle PPNR (between −2 mm and −6 mm) than the upper part above −2 mm (*P* = 0.012) and *A* Bottom: coherence with ROI 3 was higher in the lower PPNR (below −6 mm) than the upper PPNR above −2 mm (*P* = 0.008). Error bars represent SEM. The coherence spectra at each height are shown in *B*. Data are shown as mean (heavy line) and SEM (lighter shaded area). Absolute coherence is shown in black and is maximal in the middle PPNR with ROI 2 and the lower PPNR with ROI 3. To investigate whether coherence represents spurious volume conduction, we also calculated imaginary coherence spectra which are shown in green. Imaginary coherence is maximal in the same regions as absolute coherence suggesting that these are not spurious findings. Note that peak coherence shown in *B* is slightly higher than mean coherence across the alpha band shown in *A*.

**Figure 5.**
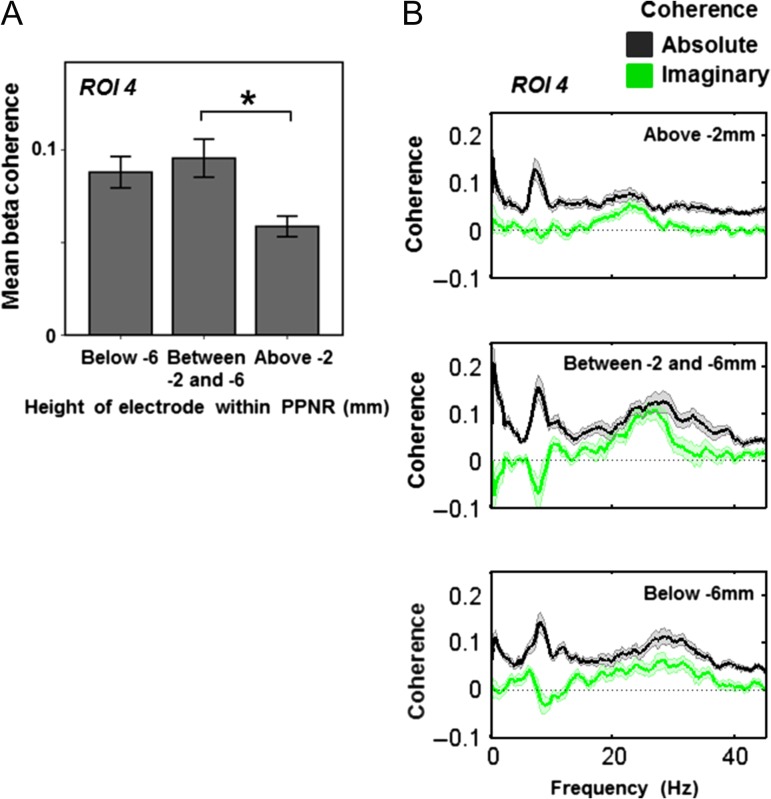
Beta coherence varies within the PPNR. Source activity was extracted from the peak voxel of beta coherence (ROI 4, medial motor strip and neighboring areas), taken from the previous SPM analysis in the medial cingulate. Mean beta coherence was then calculated and the resulting values were subjected to a mixed-effects ANOVA, with subject as a random factor and dopamine (ON vs. OFF) and electrode height as factors of interest. Electrode height within the PPNR was split into three groups as described in the legend to Fig. 4. Mean beta coherence as a function of electrode height is shown in *A*. The main effect of height was significant and post hoc *t-*tests showed that coherence was higher in the middle PPNR (between −2 and −6 mm) than the upper part (*P* = 0.015). The coherence spectra at each height are shown in *B*. Data are shown as mean (heavy line) and SEM (lighter shaded area). Absolute coherence is shown in black and in the beta frequency range this is maximal in the middle PPNR. To investigate whether coherence represents spurious volume conduction, we also calculated imaginary coherence spectra which are shown in green. Again, imaginary beta band coherence is maximal in the middle PPNR, suggesting that this is not a spurious finding. Note that the peak beta coherence shown in *B* is slightly higher than mean coherence across the beta band shown in *A*.

The peak locations of alpha and beta coherence were taken forward for further analysis. Source activity was extracted at these four locations in all subjects using LCMV beamforming. Coherence and imaginary coherence was calculated between the source activity and the PPNR-LFP. The mean coherence across the frequency range of interest (8–12 Hz for alpha ROIs 1–3 and 18–33 Hz for beta ROI 4) was then calculated and the resulting coherence values were subjected to a mixed-effects ANOVA (individually for each ROI), with subject as a random factor and dopamine (ON vs. OFF) and electrode height (above −2 mm, between −2 and −6 mm, below −6 mm) as fixed factors. Results are presented individually for each ROI below (see also Figs 4 and 5).

*ROI 1 (subgenual cingulate; alpha band)*: there was no significant main effect of electrode height (*F*(2,57) = 1.56, *P* = 0.219) or dopamine (*F*(1,57) = 0.818, *P* = 0.369) and no significant interaction effect (*F*(2,57) = 1.04, *P* = 0.359) on PPNR coherence with ROI 1.

*ROI 2 (posterior midcingulate; alpha band)*: there was a significant main effect of electrode height (*F*(2,57) = 5.06, *P* = 0.010), but no significant effect of dopamine (*F*(1,57) = 2.23, *P* = 0.141) and no significant interaction effect (*F*(2,57) = 0.29, *P* = 0.747) on PPNR coherence with ROI 2. Post hoc *t*-tests revealed a higher alpha band coherence between −2 and −6 mm height than above −2 mm (*P* = 0.012 after Bonferroni correction) (see Fig. 4).

*ROI 3 (brainstem, mostly dorsal pons; alpha band)*: there was a significant main effect of electrode height (*F*(2,57) = 4.98, *P* = 0.010), but no significant effect of dopamine (*F*(1,57) = 1.58, *P* = 0.214) and no significant interaction effect (*F*(2,57) = 0.05, *P* = 0.950) on PPNR coherence with ROI 3. Post hoc *t*-tests revealed a higher alpha band coherence below −6 mm height than above −2 mm (*P* = 0.008 after Bonferroni correction) (see Fig. 4).

*ROI 4 (medial motor strip and neighbouring areas; beta band)*: there was a significant main effect of electrode height (*F*(2,57) = 4.93, *P* = 0.011), but no significant effect of dopamine (*F*(1,57) = 0.82, *P* = 0.369) and no significant interaction effect (*F*(2,57) = 0.256, *P* = 0.775) on PPNR coherence with ROI 4. Post hoc *t*-tests revealed a higher beta band coherence between −2 and −6 mm height than above −2 mm (*P* = 0.015 after Bonferroni correction) (see Fig. 5).

Therefore, the main finding was that electrode height within the PPNR significantly affected coherence with ROIs 2 and 3 in the alpha frequency range and ROI 4 in the beta frequency range. We, therefore, present the mean coherence (Figs 4*A* and 5*A*) and the mean coherence spectra (Figs 4*B* and 5*B*, black lines) at each electrode height division in ROIs where height effects were significant (ROIs 2–4, ROI 1 not shown). Finally, to demonstrate that coherence with the ROIs was not due to spurious volume conduction, we calculated imaginary coherence (Figs 4*B* and 5*B*, black lines). Imaginary coherence implies a delay in information transfer between the two signals and as such it cannot be due to confounding factors such as volume conduction. Further post hoc statistical testing of imaginary coherence would be difficult to perform in an unbiased way (we have already detected significant coherence in this area) given our low number of subjects. However, visual inspection of the imaginary coherence spectra suggests that there is substantial imaginary coherence specific to the height and frequency range where significant effects on coherence have been detected. Specifically, imaginary alpha (but not beta) coherence with ROIs 2 and 3 is maximal below −2 mm, whilst imaginary beta (but not alpha) coherence with ROI 4 is maximal below −2 mm (Figs 4*B* and 5*B*, green lines). This supports our findings, further suggesting that they are due to genuine connectivity, rather than volume conduction.

It is difficult to estimate the individual variability of source location and frequency in this small and heterogeneous cohort, however by finding the closest individual DICS peaks to each ROI described above, we were still able to broadly demonstrate the division of dorsal beta areas with more anterior alpha areas (see Supplementary Fig. S1).

## Discussion

We have shown that in PD patients at rest, the PPNR is functionally coupled with distinct brain regions. Coupling occurred in the alpha (8–12 Hz) and beta (18–33 Hz) bands, forming two spatially and spectrally distinct resting networks (RNs). We note that this should not be confused with classical fMRI-based resting-state networks, which are detected in a similar behavioral state (awake but resting) but which rely on much slower fluctuations (usually <0.1 Hz) in blood oxygenation that are assumed to be a downstream consequence of neuronal activity (Raichle et al. 2001; Fransson 2005). The alpha band RN has three major hubs: the subgenual cingulate (also termed sub-callosal cingulate), the posterior midcingulate, and the brainstem (predominantly dorsal pons). The beta band RN has one dominant hub in the SMA/posterior midcingulate, placed more posteriorly within the cingulum than the nearby alpha hub. This broad distinction into two networks points to the functional position of the PPN at the junction between motor cortical planning areas and areas implicated in arousal. The extent of functional coupling with the PPNR was remarkable, given the evidence of partial loss of cholinergic neurons in the PPN pars compacta, in PD (Jellinger 1999; Manaye et al. 1999).

Before discussing the possible implications of these findings, we should outline some of the important limitations of this study. Firstly, we studied a relatively small number of relatively heterogeneous subjects. However, given the scarcity of human PPNR functional connectivity data and the high fidelity of direct human brainstem electrode recordings (as opposed to other functional techniques such as fMRI), we believe these results are still valuable. Secondly, these data are susceptible to confounds related to the use of different MEG systems and amplifiers, and due to ferromagnetic artefact from DBS electrodes. However, by using techniques such as spatial filtering (beamforming), signal space separation and spatial normalization, we were able to obtain comparable coherence estimates between subjects and conditions (Litvak et al. 2010). Similar techniques have been previously used by us to explore the functional connectivity of the subthalamic nucleus with findings that are consistent with those of other studies using the same or variations of this approach (Hirschmann et al. 2011, Litvak et al. 2011a; Oswal et al. 2013, 2016a). Finally, the intracranial electrode locations were heterogeneous and inevitably some of the intracranial electrodes contacts were outside the presumed PPN, lying in nearby structures. Therefore, we have initially reported connectivity with all contacts (including those in the PPN and surrounding structures), before leveraging the variation in electrode location to contrast connectivity between different rostro-caudal subregions.

### Spatial Topography of the Alpha and Beta PPNR Networks

Our study identified patterns of frequency-dependent functional connectivity and although in this study, we cannot distinguish whether the underlying anatomical connections are monosynaptic or polysynaptic, it is still interesting to frame our functional connectivity data within the currently understood neuroanatomical connectivity of the PPN and its surrounding structures.

The alpha frequency RN identified here is centred around the cingulate and dorsal pons. The most anterior region (ROI1, Fig. 3) overlaps with the subgenual cingulate which has been shown to modulate mood (specifically negative valance emotions such as sadness) and autonomic arousal (Vogt 2005; Ressler and Mayberg 2007) and interestingly this region is itself an experimental target for DBS for depression (Ressler and Mayberg 2007). This region is not known to directly synapse with the PPN and so we should entertain several possible interpretations. Firstly, the result may be a false positive finding. Alternatively, it could be genuine and represent connectivity with neighboring nuclei to the PPN such as the parabrachial nucleus, which the subgenual cingulate is known to project to (Vogt 2005). Alternatively, the functional connectivity we have detected in the alpha band may be via an intermediate invisible node, such as the central nucleus of the amygdala, which has connections to both the subgenual cingulate and a number of brainstem nuclei including the PPN itself, as well as the nearby lateral dorsal tegmental nucleus (Davis 1992; Semba and Fibiger 1992; Vogt 2005). It is interesting to speculate a link between the PPNR and the cortical–subcortical network linked to fear and arousal, as this pathway has been implicated in behavioral freezing in the context of fear (Kalin et al. 2004), which shares some similarities to the pathological freezing of gait in our PD cohort.

The second alpha node was located in the posterior midcingulate (ROI 2, Fig. 3), also termed the “motor” region of the cingulate. This region has been linked to integrating sensorimotor feedback during movement (Vogt 2005). A number of locations within the medial frontal wall (including the cingulate) are known to project to the PPNR, in particular to the pars dissipata of the PPN itself (Sesack et al. 1989; Alderson et al. 2008). This is consistent with our results, which show that alpha coherence with the cingulate was greater in the middle PPNR (between −2 and −6 mm height relative to the PM line, that is, the level of the pars dissipata of the PPN) rather than the upper PPNR.

The final node of the alpha network was located within the posterior brainstem and maximal in the dorsal pons (ROI 3, Fig. 3). The exact location remains unclear given that the resolution of MEG in the brainstem/cerebellum is poor. However, MEG has been shown to be sensitive to brainstem activity such as auditory brainstem responses (Parkkonen et al. 2009), and indeed, brainstem alpha coherence has also been seen with the subthalamic nucleus (STN) region in patients with PD (Litvak et al. 2011a), and with EMG in patients with tremor (Schnitzler et al. 2009). The potential location identified by our analysis overlaps with the location of the PPN itself raising the possibility that it may in fact represent the MEG picking-up signal from the PPN. However, this is unlikely to account for all of the activity detected because imaginary coherence between this region and the PPNR electrodes was consistently present, suggesting that the detected alpha-coupled region may be another brainstem nucleus or even the cerebellar vermis. Consistent with this, we found that coherence was maximal with the lower PPNR (as compared to the upper PPNR), which is outside the presumed PPN proper.

The beta frequency RN was dominated by a single node in the medial frontal wall that overlaps with the supplementary area (SMA), the leg area of the primary motor cortex and the posterior midcingulate. These areas are critical to the planning and execution of movement, and are particularly suited to potentially modulate gait. Parts of the motor cortex and medial frontal wall directly project to the pars dissipata of the PPN (Sesack et al. 1989; Muthusamy et al. 2007).

The extensive patterns of cortical coupling with the PPNR described above are in keeping with this region's rich direct and indirect, polysynaptic connectivity (Sesack et al. 1989; Hazrati and Parent 1992; Pahapill 2000; Mena-Segovia et al. 2004; Aravamuthan et al. 2008; Schofield and Motts 2009; Alam et al. 2011; Martinez-Gonzalez et al. 2011), but they do not represent a complete picture of it. Rodent studies increasingly favor a rostral-caudal gradient of connectivity along the PPN (Martinez-Gonzalez et al. 2011; Valencia et al. 2014; Gut and Winn 2015), and if we assume a similar gradient in humans, then the cortical nodes identified in this functional connectivity analysis are primarily known to synapse onto the *caudal* PPN. In particular, *rostral* PPN afferents from the basal ganglia (via the Substantia Nigra Pars Reticulata and Globus Pallidus Interna) and rostral PPN efferents to the whole substantia nigra complex and hypothalamus are conspicuous in their absence. This may be explained by a number of potential technical reasons—MEG sensors are more sensitive to surface structures (nearer to the sensors) and to layered structures such as cortex rather than nuclei (summed postsynaptic potentials are stronger if cell dendrites are better aligned). However, cortical areas known to input into the caudal PPN such as the motor cortex and mid-cingulate *are* described in our analysis. It is unclear what structure the most caudal - alpha node represents but it is interesting to note the known PPN connections it could represent, such as those with the cerebellum, dorsal raphe, and colliculi are all suggested to synapse with the entire PPN axis or the caudal region preferentially. Therefore it seems that, using the current MEG-based methodology, the most easily detectable sources coupled to the PPN, preferentially couple to the caudal PPN. One exception is the STN, which is poorly detected by MEG given it is such a small deep nucleus. The previous work of our group and others (Hirschmann et al. 2011; Litvak et al. 2011a) combining MEG with STN recordings showed no evidence of direct pickup of STN signal by MEG. It is, therefore, not surprising that STN does not appear in the results of the current analysis and these results do not rule out the existence of coherence between PPNR and the STN that could be detected by simultaneous LFP recording from the two structures.

### Comparison to Cortico-subthalamic Networks

It is interesting to note that the cortical and subcortical topographies of the RNs characterized by activities in the alpha and beta bands share some similarity with those with similar spectral organization demonstrated to be coupled with the STN in PD patients (Hirschmann et al. 2011; Litvak et al. 2011a), underscoring the functional interdependency of the PPNR and STN. Peak beta coherence is seen between the medial motor strip and neighboring areas and both the PPNR and STN—and in both regions the peak frequency is around 30 Hz [Fig. 5 and see (Litvak et al. 2011a)]. Additionally strong alpha coherence with a brainstem region is seen with both the STN and PPNR. However, it is important to acknowledge that although some features of the pattern of distributed connectivity may be broadly similar between these two subcortical hubs there are also some differences. Specifically, no coherence between the PPNR and temporoparietal region was demonstrated in contrast to the STN coupling with this area (Hirschmann et al. 2011; Litvak et al. 2011a), and no alpha coherence was detected between the subgenual cingulate and the STN in contrast to the present data. In addition, beta coherence with the STN extended more anteriorly and laterally than did beta coherence with the PPNR, involving the pre-SMA and the dorsolateral prefrontal cortex.

### Possible Function of the Alpha and Beta Networks

The medial frontal regions coupled with the PPNR at rest have been shown to be modulated by gait or differ in activity between PD patients with and without freezing of gait—the major symptom in our cohort (reviewed in Bartels and Leenders 2008). For example, PD patients show altered gait-related perfusion in the SMA and cingulate cortex (Hanakawa et al. 1999; Nutt et al. 2011). It is interesting to note that we have identified a corresponding set of functional connections between these areas and a key locomotor region, the PPNR, suggesting that the PPNR may act as a hub through which these cortical effects are co-ordinated. Indeed, low frequency stimulation of the PPNR alters resting cortical glucose metabolism and cerebral blood flow in a large network of areas that include the cingulate, prefrontal areas, temporal lobe, thalamus, and cerebellum (Ballanger et al. 2009; Ceravolo et al. 2011).

What specialized functions might the RNs possibly mediate? The alpha frequency band has often been associated with attention and the allocation of processing resources (Palva and Palva 2007), and we have previously speculated that the alpha band RN with the STN may subserve attentional functions in patients with PD (Litvak et al. 2011a). The latter may be particularly critical in the present patient group which was preselected for the prominence of gait difficulties, particularly freezing. A relationship between attentional control and gait performance is increasingly recognized (Yarnall et al. 2011). Gait speed reduces in healthy subjects, elderly fallers and in PD during the performance of a second, unrelated task (“dual tasking”) (Hausdorff et al. 2003; Springer et al. 2006; Lamoth et al. 2011). Dual tasking and other processes that divert attention away from walking can all also precipitate gait freezing (Springer et al. 2006). In PD, attentional deficits are increased in patients that fall (Allcock et al. 2009) and freeze during gait (Amboni et al. 2008; Yogev-Seligmann et al. 2008).

The PPN is considered a component of the “reticular activating system” and may modulate states of arousal and attention (Winn 2006). In line with such a role, PPN stimulation in patients with PD may increase rapid eye movement sleep (Romigi et al. 2008; Lim et al. 2009) and diurnal vigilance (Ferraye et al. 2010), and PPNR-cortical EEG coherence has been reported in the alpha band during wakefulness (Androulidakis et al. 2008). We suggest that the PPNR may be another subcortical relay in attentional networks characterized by oscillatory activity in the alpha frequency band (Litvak et al. 2011a; Thevathasan et al. 2012). Given the functional and anatomical heterogeneity of the PPNR, and the spread of electrodes within it, one may have expected a large variation in coupling due to electrode position within the PPNR. Indeed the middle PPNR (including the caudal PPN), between −2 and −6 mm relative to the PM line, may be particularly important in supporting a possible role in gait control through attentional modulation. Local oscillatory synchrony in the alpha band was maximal here and has been shown to correlate with gait performance (Thevathasan et al. 2012) but also visual cue presentation and gait imagination (Lau et al. 2015) suggesting it is correlated with attentional control rather than motor performance. Consistent with the special importance of this middle section of the PPNR, this region showed the greatest coherence with the midcingulate in both alpha and beta frequency bands (Figs 4 and 5). We have previously argued that this middle section of the PPNR includes the caudal part of the “pars dissipata” of the PPN (Thevathasan et al. 2012). The pars dissipata of the PPN, as defined by immunohistochemical labeling of choline-acetyltransferase in humans, extends both rostrally and caudally from the central pars compacta, (Mesulam et al. 1989; Manaye et al. 1999), and has been implicated in gait and its dysfunction (Karachi et al. 2010). It should, however, be acknowledged that the pars dissipata of the PPN has indistinct boundaries, and of note, just medial to its caudal boundary is the laterodorsal tegmental nucleus which is also rich in cholinergic neurons (Manaye et al. 1999). In addition, unlike alpha LFP activity, neuronal firing in the PPN associated with imagined gait is more diffuse (Tattersall et al. 2014).

Still, it must be stressed that the association of the PPNR alpha band RN with attentional function remains speculative and requires future corroboration. Nor does it discount the possibility that the beta band RN may also play a role in attention, possibly through its functional connectivity with the medial cingulate. Thus, it has been suggested that the medial frontal cortex is involved in compensation for low-arousal states where attention is still necessary, such as in monotonous vigilance tasks like walking (Portas et al. 1998; Coull et al. 2004; Gilbert et al. 2006; Fischer et al. 2008). This compensation has been linked to enhanced frontal power in the beta frequency band (Fischer et al. 2008)—precisely the frequency of dominant connectivity between midline frontal regions and the PPNR. Of course task-related modulation of activity in the beta RN, with its extensive connectivity to motor areas, may also contribute to more explicitly motor aspects of gait. Consistent with this, both beta band LFP power in the PPNR, and beta coherence between the medial frontal cortex and the PPNR are reactive to movement in patients with PD (Tsang et al. 2010; Lau et al. 2015).

## Conclusions

Our results support the notion of two principal RNs involving the PPNR, which may be distinguished in terms of their spectral tuning and their wider connectivity. This represents an initial step in determining the different functional associations of these circuits which, here are characterized only at rest. Future work may help to validate the behavioral function of these networks, in particular, whether the contrasting alpha- and beta-tuned RNs may reflect arousal/attentional and motor planning networks, respectively, and whether they are modulated during walking or imagined gait. Eventually, it may be possible to differentially modulate these systems through stimulation, by harnessing their different resonance properties and any fine scale differences in their organization within the PPNR.

## Supplementary Material

Supplementary Data

Supplementary Data

## Notes

We would like to thank David Bradbury of the Wellcome Trust Centre for Neuroimaging and Sven Braeutigam and Anling Rao of the Oxford Centre for Human Brain Activity for their assistance conducting the experiments. *Conflict of Interest*: None declared.

## References

[bhw340C1] Alam M, Schwabe K, Krauss JK 2011 The pedunculopontine nucleus area: critical evaluation of interspecies differences relevant for its use as a target for deep brain stimulation. Brain. 134:11–23. 21147837 10.1093/brain/awq322

[bhw340C2] Alderson HL, Latimer MP, Winn P 2008 A functional dissociation of the anterior and posterior pedunculopontine tegmental nucleus: excitotoxic lesions have differential effects on locomotion and the response to nicotine. Brain Struct Funct. 213:247–253. 18266007 10.1007/s00429-008-0174-4 PMC2522332

[bhw340C3] Allcock LM, Rowan EN, Steen IN, Wesnes K, Kenny RA, Burn DJ 2009 Impaired attention predicts falling in Parkinson's disease. Parkinsonism Relat Disord. 15:110–115. 18487069 10.1016/j.parkreldis.2008.03.010

[bhw340C4] Amboni M, Cozzolino A, Longo K, Picillo M, Barone P 2008 Freezing of gait and executive functions in patients with Parkinson's disease. Mov Disord. 23:395–400. 18067193 10.1002/mds.21850

[bhw340C5] Androulidakis AG, Mazzone P, Litvak V, Penny W, Dileone M, Gaynor LMFD, Tisch S, Di Lazzaro V, Brown P 2008 Oscillatory activity in the pedunculopontine area of patients with Parkinson's disease. Exp Neurol. 211:59–66. 18282571 10.1016/j.expneurol.2008.01.002

[bhw340C6] Aravamuthan BR, Stein JF, Aziz TZ 2008 The anatomy and localization of the pedunculopontine nucleus determined using probabilistic diffusion tractography [corrected]. Br J Neurosurg. 22(Suppl 1):S25–S32. 19085350 10.1080/02688690802448251

[bhw340C7] Arnulf I, Ferraye M, Fraix V, Benabid AL, Chabardès S, Goetz L, Pollak P, Debû B 2010 Sleep induced by stimulation in the human pedunculopontine nucleus area. Ann Neurol. 67:546–549. 20437591 10.1002/ana.21912

[bhw340C8] Ballanger B, Lozano AM, Moro E, van Eimeren T, Hamani C, Chen R, Cilia R, Houle S, Poon YY, Lang AE, et al 2009 Cerebral blood flow changes induced by pedunculopontine nucleus stimulation in patients with advanced Parkinson's disease: a [(15)O] H2O PET study. Hum Brain Mapp. 30:3901–3909. 19479730 10.1002/hbm.20815 PMC6871082

[bhw340C9] Bartels AL, Leenders KL 2008 Brain imaging in patients with freezing of gait. Mov Disord. 23(Suppl 2):S461–S467. 18668627 10.1002/mds.21912

[bhw340C10] Bloem BR, Hausdorff JM, Visser JE, Giladi N 2004 Falls and freezing of gait in Parkinson's disease: a review of two interconnected, episodic phenomena. Mov Disord. 19:871–884. 15300651 10.1002/mds.20115

[bhw340C11] Boeve BF, Silber MH, Saper CB, Ferman TJ, Dickson DW, Parisi JE, Benarroch EE, Ahlskog JE, Smith GE, Caselli RC, et al 2007 Pathophysiology of REM sleep behaviour disorder and relevance to neurodegenerative disease. Brain. 130:2770–2788. 17412731 10.1093/brain/awm056

[bhw340C12] Buzsaki G, Draguhn A 2004 Neuronal oscillations in cortical networks. Science. 304:1926–1929. 15218136 10.1126/science.1099745

[bhw340C13] Ceravolo R, Brusa L, Galati S, Volterrani D, Peppe A, Siciliano G, Pierantozzi M, Moschella V, Bonuccelli U, Stanzione P, et al 2011 Low frequency stimulation of the nucleus tegmenti pedunculopontini increases cortical metabolism in parkinsonian patients. Eur J Neurol. 18:842–849. 21087362 10.1111/j.1468-1331.2010.03254.x

[bhw340C14] Chen J, Nakamura M, Kawamura T, Takahashi T, Nakahara D 2006 Roles of pedunculopontine tegmental cholinergic receptors in brain stimulation reward in the rat. Psychopharmacology (Berl). 184:514–522. 16385418 10.1007/s00213-005-0252-8

[bhw340C15] Classen J, Schnitzler A 2010 What does the pedunculopontine nucleus do. Neurology. 75:944–945. 20702789 10.1212/WNL.0b013e3181f25b73

[bhw340C16] Coull JT, Vidal F, Nazarian B, Macar F 2004 Functional anatomy of the attentional modulation of time estimation. Science. 303:1506–1508. 15001776 10.1126/science.1091573

[bhw340C17] Davis M 1992 The role of the amygdala in fear and anxiety. Annu Rev Neurosci. 15:353–375. 1575447 10.1146/annurev.ne.15.030192.002033

[bhw340C18] Ferraye MU, Debû B, Fraix V, Goetz L, Ardouin C, Yelnik J, Henry-Lagrange C, Seigneuret E, Piallat B, Krack P, et al 2010 Effects of pedunculopontine nucleus area stimulation on gait disorders in Parkinson's disease. Brain. 133:205–214. 19773356 10.1093/brain/awp229

[bhw340C19] Fischer T, Langner R, Birbaumer N, Brocke B 2008 Arousal and attention: self-chosen stimulation optimizes cortical excitability and minimizes compensatory effort. J Cogn Neurosci. 20:1443–1453. 18303981 10.1162/jocn.2008.20101

[bhw340C20] Foltynie T, Hariz MI 2010 Surgical management of Parkinson's disease. Expert Rev Neurother. 10:903–914. 20518607 10.1586/ern.10.68

[bhw340C21] Fransson P 2005 Spontaneous low-frequency BOLD signal fluctuations: An fMRI investigation of the resting-state default mode of brain function hypothesis. Hum Brain Mapp. 26:15–29. 15852468 10.1002/hbm.20113 PMC6871700

[bhw340C22] Garcia-Rill E 1991 The pedunculopontine nucleus. Prog Neurobiol. 36:363–389. 1887068 10.1016/0301-0082(91)90016-t

[bhw340C23] Garcia-Rill E, Houser CR, Skinner RD, Smith W, Woodward DJ 1987 Locomotion-inducing sites in the vicinity of the pedunculopontine nucleus. Brain Res Bull. 18:731–738. 3304544 10.1016/0361-9230(87)90208-5

[bhw340C24] Gaynor LM, Kühn AA, Dileone M, Litvak V, Eusebio A, Pogosyan A, Androulidakis AG, Tisch S, Limousin P, Insola A, et al 2008 Suppression of beta oscillations in the subthalamic nucleus following cortical stimulation in humans. Eur J Neurosci. 28:1686–1695. 18657185 10.1111/j.1460-9568.2008.06363.x PMC2695156

[bhw340C25] Giladi N, McDermott MP, Fahn S, Przedborski S, Jankovic J, Stern M, Tanner C 2001 Freezing of gait in PD: prospective assessment in the DATATOP cohort. Neurology. 56:1712–1721. 11425939 10.1212/wnl.56.12.1712

[bhw340C26] Giladi N, Shabtai H, Simon E, Biran S, Tal J, Korczyn A 2000 Construction of freezing of gait questionnaire for patients with Parkinsonism. Parkinsonism Relat Disord. 6:165–170. 10817956 10.1016/s1353-8020(99)00062-0

[bhw340C27] Giladi N, Tal J, Azulay T, Rascol O, Brooks DJ, Melamed E, Oertel W, Poewe WH, Stocchi F, Tolosa E 2009 Validation of the freezing of gait questionnaire in patients with Parkinson's disease. Mov Disord. 24:655–661. 19127595 10.1002/mds.21745

[bhw340C28] Gilbert SJ, Simons JS, Frith CD, Burgess PW 2006 Performance-related activity in medial rostral prefrontal cortex (area 10) during low-demand tasks. J Exp Psychol Hum Percept Perform. 32:45–58. 16478325 10.1037/0096-1523.32.1.45

[bhw340C29] Gross J, Kujala J, Hamalainen M, Timmermann L, Schnitzler A, Salmelin R 2001 Dynamic imaging of coherent sources: Studying neural interactions in the human brain. Proc Natl Acad Sci USA. 98:694–699. 11209067 10.1073/pnas.98.2.694 PMC14650

[bhw340C30] Gut NK, Winn P 2015 Deep brain stimulation of different pedunculopontine targets in a novel rodent model of parkinsonism. J Neurosci. 35:4792–4803. 25810510 10.1523/JNEUROSCI.3646-14.2015 PMC4389588

[bhw340C31] Hallett M 2008 The intrinsic and extrinsic aspects of freezing of gait. Mov Disord. 23(Suppl 2):S439–S443. 18668625 10.1002/mds.21836 PMC4758454

[bhw340C32] Hanakawa T, Katsumi Y, Fukuyama H, Honda M, Hayashi T, Kimura J, Shibasaki H 1999 Mechanisms underlying gait disturbance in Parkinson's disease: a single photon emission computed tomography study. Brain. 122(Pt 7):1271–1282. 10388793 10.1093/brain/122.7.1271

[bhw340C33] Hausdorff JM, Balash J, Giladi N 2003 Effects of cognitive challenge on gait variability in patients with Parkinson's disease. J Geriatr Psychiatry Neurol. 16:53–58. 12641374 10.1177/0891988702250580

[bhw340C34] Hazrati LN, Parent A 1992 Projection from the deep cerebellar nuclei to the pedunculopontine nucleus in the squirrel monkey. Brain Res. 585:267–271. 1380869 10.1016/0006-8993(92)91216-2

[bhw340C35] Hirschmann J, Hartmann CJ, Butz M, Hoogenboom N, Ozkurt TE, Elben S, Vesper J, Wojtecki L, Schnitzler A 2013a. A direct relationship between oscillatory subthalamic nucleus-cortex coupling and rest tremor in Parkinson's disease. Brain. 136:3659–3670. 24154618 10.1093/brain/awt271

[bhw340C36] Hirschmann J, Özkurt TE, Butz M, Homburger M, Elben S, Hartmann CJ, Vesper J, Wojtecki L, Schnitzler A 2011 Distinct oscillatory STN-cortical loops revealed by simultaneous MEG and local field potential recordings in patients with Parkinson's disease. Neuroimage. 55:1159–1168. 21122819 10.1016/j.neuroimage.2010.11.063

[bhw340C37] Hirschmann J, Özkurt TE, Butz M, Homburger M, Elben S, Hartmann CJ, Vesper J, Wojtecki L, Schnitzler A 2013b. Differential modulation of STN-cortical and cortico-muscular coherence by movement and levodopa in Parkinson's disease. Neuroimage. 68:203–213. 23247184 10.1016/j.neuroimage.2012.11.036

[bhw340C38] Jankovic J 2008 Parkinson's disease: clinical features and diagnosis. J Neurol Neurosurg Psychiatry. 79:368–376. 18344392 10.1136/jnnp.2007.131045

[bhw340C39] Jellinger KA 1999 Post mortem studies in Parkinson's disease--is it possible to detect brain areas for specific symptoms. J Neural Transm Suppl. 56:1–29. 10370901 10.1007/978-3-7091-6360-3_1

[bhw340C40] Kalin NH, Shelton SE, Davidson RJ 2004 The role of the central nucleus of the amygdala in mediating fear and anxiety in the primate. J Neurosci. 24:5506–5515. 15201323 10.1523/JNEUROSCI.0292-04.2004 PMC6729317

[bhw340C41] Karachi C, Grabli D, Bernard FA, Tandé D, Wattiez N, Belaid H, Bardinet E, Prigent A, Nothacker H-P, Hunot S, et al 2010 Cholinergic mesencephalic neurons are involved in gait and postural disorders in Parkinson disease. J Clin Invest. 120:2745–2754. 20628197 10.1172/JCI42642 PMC2912198

[bhw340C42] Kilner JM, Friston KJ 2010 Topological inference for EEG and MEG. Ann Appl Stat. 4:1272–1290.

[bhw340C43] Lamoth CJ, van Deudekom FJ, van Campen JP, Appels BA, de Vries OJ, Pijnappels M 2011 Gait stability and variability measures show effects of impaired cognition and dual tasking in frail people. J Neuroeng Rehabil. 8:2. 21241487 10.1186/1743-0003-8-2 PMC3034676

[bhw340C44] Lau B, Welter M-L, Belaid H, Fernandez Vidal S, Bardinet E, Grabli D, Karachi C 2015 The integrative role of the pedunculopontine nucleus in human gait. Brain. 138:1284–1296. 25765327 10.1093/brain/awv047 PMC5963406

[bhw340C45] Li M, Zhang W 2015 Oscillations in pedunculopontine nucleus in Parkinson's disease and its relationship with deep brain stimulation. Front Neural Circuits. 9:47. 26388741 10.3389/fncir.2015.00047 PMC4556974

[bhw340C46] Lim AS, Lozano AM, Moro E, Hamani C, Hutchison WD, Dostrovsky JO, Lang AE, Wennberg RA, Murray BJ 2007 Characterization of REM-sleep associated ponto-geniculo-occipital waves in the human pons. Sleep. 30:823–827. 17682651 10.1093/sleep/30.7.823 PMC1978372

[bhw340C47] Lim AS, Moro E, Lozano AM, Hamani C, Dostrovsky JO, Hutchison WD, Lang AE, Wennberg RA, Murray BJ 2009 Selective enhancement of rapid eye movement sleep by deep brain stimulation of the human pons. Ann Neurol. 66:110–114. 19670451 10.1002/ana.21631

[bhw340C48] Litvak V, Eusebio A, Jha A, Oostenveld R, Barnes GR, Penny WD, Zrinzo L, Hariz MI, Limousin P, Friston KJ, et al 2010 Optimized beamforming for simultaneous MEG and intracranial local field potential recordings in deep brain stimulation patients. Neuroimage. 50:1578–1588. 20056156 10.1016/j.neuroimage.2009.12.115 PMC3221048

[bhw340C49] Litvak V, Jha A, Eusebio A, Oostenveld R, Foltynie T, Limousin P, Zrinzo L, Hariz MI, Friston K, Brown P 2011a. Resting oscillatory cortico-subthalamic connectivity in patients with Parkinson's disease. Brain. 134:359–374. 21147836 10.1093/brain/awq332

[bhw340C50] Litvak V, Mattout J, Kiebel S, Phillips C, Henson R, Kilner J, Barnes G, Oostenveld R, Daunizeau J, Flandin G, et al 2011b. EEG and MEG data analysis in SPM8. Comput Intell Neurosci. 2011:852961. 21437221 10.1155/2011/852961 PMC3061292

[bhw340C51] Magill PJ, Sharott A, Bolam JP, Brown P 2006 Delayed synchronization of activity in cortex and subthalamic nucleus following cortical stimulation in the rat. J Physiol. 574:929–946. 16709634 10.1113/jphysiol.2006.110379 PMC1817747

[bhw340C52] Manaye KF, Zweig R, Wu D, Hersh LB, De Lacalle S, Saper CB, German DC 1999 Quantification of cholinergic and select non-cholinergic mesopontine neuronal populations in the human brain. Neuroscience. 89:759–770. 10199611 10.1016/s0306-4522(98)00380-7

[bhw340C53] Martinez-Gonzalez C, Bolam JP, Mena-Segovia J 2011 Topographical organization of the pedunculopontine nucleus. Front Neuroanat. 5:22. 21503154 10.3389/fnana.2011.00022 PMC3074429

[bhw340C54] Mattout J, Henson RN, Friston KJ 2007 Canonical source reconstruction for MEG. Comput Intell Neurosci. 67613. 18350131 10.1155/2007/67613 PMC2266807

[bhw340C55] Mazzone P, Lozano A, Stanzione P, Galati S, Scarnati E, Peppe A, Stefani A 2005 Implantation of human pedunculopontine nucleus: a safe and clinically relevant target in Parkinson's disease. Neuroreport. 16:1877–1881. 16272871 10.1097/01.wnr.0000187629.38010.12

[bhw340C56] Mena-Segovia J, Bolam JP, Magill PJ 2004 Pedunculopontine nucleus and basal ganglia: distant relatives or part of the same family. Trends Neurosci. 27:585–588. 15374668 10.1016/j.tins.2004.07.009

[bhw340C57] Mesulam MM, Geula C, Bothwell MA, Hersh LB 1989 Human reticular formation: cholinergic neurons of the pedunculopontine and laterodorsal tegmental nuclei and some cytochemical comparisons to forebrain cholinergic neurons. J Comp Neurol. 283:611–633. 2545747 10.1002/cne.902830414

[bhw340C58] Mitra PP, Pesaran B 1999 Analysis of dynamic brain imaging data. Biophys J. 76:691–708. 9929474 10.1016/S0006-3495(99)77236-X PMC1300074

[bhw340C59] Morita H, Hass CJ, Moro E, Sudhyadhom A, Kumar R, Okun MS 2014 Pedunculopontine nucleus stimulation: where are we now and what needs to be done to move the field forward. Front Neurol. 5:243. 25538673 10.3389/fneur.2014.00243 PMC4255598

[bhw340C60] Moro E, Hamani C, Poon Y-Y, Al-Khairallah T, Dostrovsky JO, Hutchison WD, Lozano AM 2010 Unilateral pedunculopontine stimulation improves falls in Parkinson's disease. Brain. 133:215–224. 19846583 10.1093/brain/awp261

[bhw340C61] Moruzzi G, Magoun HW 1949 Brain stem reticular formation and activation of the EEG. Electroencephalogr Clin Neurophysiol. 1:455–473. 18421835

[bhw340C62] Muthusamy KA, Aravamuthan BR, Kringelbach ML, Jenkinson N, Voets NL, Johansen-Berg H, Stein JF, Aziz TZ 2007 Connectivity of the human pedunculopontine nucleus region and diffusion tensor imaging in surgical targeting. J Neurosurg. 107:814–820. 17937229 10.3171/JNS-07/10/0814

[bhw340C63] Nandi D, Aziz TZ, Giladi N, Winter J, Stein JF 2002 Reversal of akinesia in experimental parkinsonism by GABA antagonist microinjections in the pedunculopontine nucleus. Brain. 125:2418–2430. 12390969 10.1093/brain/awf259

[bhw340C64] Nandi D, Jenkinson N, Stein J, Aziz T 2008 The pedunculopontine nucleus in Parkinson's disease: primate studies. Br J Neurosurg. 22(Suppl 1):S4–S8. 19085345 10.1080/02688690802448350

[bhw340C65] Nolte G, Bai O, Wheaton L, Mari Z, Vorbach S, Hallett M 2004 Identifying true brain interaction from EEG data using the imaginary part of coherency. Clin Neurophysiol. 115:2292–2307. 15351371 10.1016/j.clinph.2004.04.029

[bhw340C66] Norton AB, Jo YS, Clark EW, Taylor CA, Mizumori SJ 2011 Independent neural coding of reward and movement by pedunculopontine tegmental nucleus neurons in freely navigating rats. Eur J Neurosci. 33:1885–1896. 21395868 10.1111/j.1460-9568.2011.07649.x PMC3095748

[bhw340C67] Nutt JG, Bloem BR, Giladi N, Hallett M, Horak FB, Nieuwboer A 2011 Freezing of gait: moving forward on a mysterious clinical phenomenon. Lancet Neurol. 10:734–744. 21777828 10.1016/S1474-4422(11)70143-0 PMC7293393

[bhw340C68] Olszewski J, Baxter D 1982 The pedunculopontine nucleus (PPN), located at the junction between the midbrain and pons, is comprised of cholinergic, glutamatergic and GABAergic cells (Wang & Morales, 2009) and has been implicated in locomotion (Garcia-Rill et al. 1987; Skinner et al. 2nd ed.) Basel: Karger.

[bhw340C69] Oostenveld R, Fries P, Maris E, Schoffelen J-M 2011 FieldTrip: Open source software for advanced analysis of MEG, EEG, and invasive electrophysiological data. Comput Intell Neurosci. 2011:156869. 21253357 10.1155/2011/156869 PMC3021840

[bhw340C70] Oswal A, Brown P, Litvak V 2013 Movement related dynamics of subthalmo-cortical alpha connectivity in Parkinson's Disease. Neuroimage. 70:132–142. 23277109 10.1016/j.neuroimage.2012.12.041 PMC3591253

[bhw340C71] Oswal A, Jha A, Neal S, Reid A, Bradbury D, Aston P, Limousin P, Foltynie T, Zrinzo L, Brown P, et al 2016a. Analysis of simultaneous MEG and intracranial LFP recordings during Deep Brain Stimulation: a protocol and experimental validation. J Neurosci Methods. 261:29–46. 26698227 10.1016/j.jneumeth.2015.11.029 PMC4758829

[bhw340C72] Oswal A, Jha A, Neal S, Reid A, Bradbury D, Limousin P, Foltynie T, Zrinzo L, Hariz M, Brown P, et al 2016b. Analysis of simultaneous MEG and intracranial LFP recordings during Deep Brain Stimulation: a protocol and experimental validation. J Neurosci Methods. 261:29–46. 26698227 10.1016/j.jneumeth.2015.11.029 PMC4758829

[bhw340C73] Pahapill PA 2000 The pedunculopontine nucleus and Parkinson's disease. Brain. 123:1767–1783. 10960043 10.1093/brain/123.9.1767

[bhw340C74] Palva S, Palva JM 2007 New vistas for alpha-frequency band oscillations. Trends Neurosci. 30:150–158. 17307258 10.1016/j.tins.2007.02.001

[bhw340C75] Parkkonen L, Fujiki N, Makela JP 2009 Sources of auditory brainstem responses revisited: contribution by magnetoencephalography. Hum Brain Mapp. 30:1772–1782. 19378273 10.1002/hbm.20788 PMC6870971

[bhw340C76] Pereira EA, Muthusamy KA, De Pennington N, Joint CA, Aziz TZ 2008 Deep brain stimulation of the pedunculopontine nucleus in Parkinson's disease. Preliminary experience at Oxford. Br J Neurosurg. 22(Suppl 1):S41–S44. 19085352 10.1080/02688690802448335

[bhw340C77] Plaha P, Gill SS 2005 Bilateral deep brain stimulation of the pedunculopontine nucleus for Parkinson's disease. Neuroreport. 16:1883–1887. 16272872 10.1097/01.wnr.0000187637.20771.a0

[bhw340C78] Portas CM, Rees G, Howseman AM, Josephs O, Turner R, Frith CD 1998 A specific role for the thalamus in mediating the interaction of attention and arousal in humans. J Neurosci. 18:8979–8989. 9787003 10.1523/JNEUROSCI.18-21-08979.1998 PMC6793555

[bhw340C79] Raichle ME, MacLeod AM, Snyder AZ, Powers WJ, Gusnard DA, Shulman GL 2001 A default mode of brain function. Proc Natl Acad Sci USA. 98:676–682. 11209064 10.1073/pnas.98.2.676 PMC14647

[bhw340C80] Rappelsberger P, Petsche H 1988 Probability mapping: power and coherence analyses of cognitive processes. Brain Topogr. 1:46–54. 3274962 10.1007/BF01129339

[bhw340C81] Ressler KJ, Mayberg HS 2007 Targeting abnormal neural circuits in mood and anxiety disorders : from the laboratory to the clinic. Nat Neurosci. 10:1116–1124. 17726478 10.1038/nn1944 PMC2444035

[bhw340C82] Romigi A, Placidi F, Peppe A, Pierantozzi M, Izzi F, Brusa L, Galati S, Moschella V, Marciani MG, Mazzone P, et al 2008 Pedunculopontine nucleus stimulation influences REM sleep in Parkinson's disease. Eur J Neurol. 15:e64–e65. 18484989 10.1111/j.1468-1331.2008.02167.x

[bhw340C83] Schnitzler A, Munks C, Butz M, Timmermann L, Gross J 2009 Synchronized brain network associated with essential tremor as revealed by magnetoencephalography. Mov Disord. 24:1629–1635. 19514010 10.1002/mds.22633

[bhw340C84] Schofield BR, Motts SD 2009 Projections from auditory cortex to cholinergic cells in the midbrain tegmentum of guinea pigs. Brain Res Bull. 80:163–170. 19576264 10.1016/j.brainresbull.2009.06.015 PMC2731009

[bhw340C85] Semba K, Fibiger HC 1992 Afferent connections of the laterodorsal and the pedunculopontine tegmental nuclei in the rat: a retro- and antero-grade transport and immunohistochemical study. J Comp Neurol. 323:387–410. 1281170 10.1002/cne.903230307

[bhw340C86] Sesack SR, Deutch AY, Roth RH, Bunney BS 1989 Topographical organization of the efferent projections of the medial prefrontal cortex in the rat: an anterograde tract-tracing study with Phaseolus vulgaris leucoagglutinin. J Comp Neurol. 290:213–242. 2592611 10.1002/cne.902900205

[bhw340C87] Shen B, Nadkarni M, Zappulla RA 1999 Spectral modulation of cortical connections measured by EEG coherence in humans. Clin Neurophysiol. 110:115–125. 10348330 10.1016/s0013-4694(98)00104-7

[bhw340C88] Skinner RD, Kinjo N, Henderson V, Garcia-Rill E 1990 Locomotor projections from the pedunculopontine nucleus to the spinal cord. Neuroreport. 1:183–186. 2129877 10.1097/00001756-199011000-00001

[bhw340C89] Smith SM, Jenkinson M, Woolrich MW, Beckmann CF, Behrens TEJ, Johansen-Berg H, Bannister PR, De Luca M, Drobnjak I, Flitney DE, et al 2004 Advances in functional and structural MR image analysis and implementation as FSL. Neuroimage. 23(Suppl 1):S208–S219. 15501092 10.1016/j.neuroimage.2004.07.051

[bhw340C90] Snijders AH, Leunissen I, Bakker M, Overeem S, Helmich RC, Bloem BR, Toni I 2011 Gait-related cerebral alterations in patients with Parkinson's disease with freezing of gait. Brain. 134:59–72. 21126990 10.1093/brain/awq324

[bhw340C91] Springer S, Giladi N, Peretz C, Yogev G, Simon ES, Hausdorff JM 2006 Dual-tasking effects on gait variability: the role of aging, falls, and executive function. Mov Disord. 21:950–957. 16541455 10.1002/mds.20848

[bhw340C92] Stefani A, Lozano A, Stanzione P, Mazzone P 2007a. Targeting human PPN: few patients, numerous disputes. Brain. 130:e80–e80.

[bhw340C93] Stefani A, Lozano AM, Peppe A, Stanzione P, Galati S, Tropepi D, Pierantozzi M, Brusa L, Scarnati E, Mazzone P 2007b. Bilateral deep brain stimulation of the pedunculopontine and subthalamic nuclei in severe Parkinson's disease. Brain. 130:1596–1607. 17251240 10.1093/brain/awl346

[bhw340C94] Tattersall TL, Stratton PG, Coyne TJ, Cook R, Silberstein P, Silburn PA, Windels F, Sah P 2014 Imagined gait modulates neuronal network dynamics in the human pedunculopontine nucleus. Nat Neurosci. 17:449–454. 24487235 10.1038/nn.3642

[bhw340C95] Taulu S, Simola J 2006 Spatiotemporal signal space separation method for rejecting nearby interference in MEG measurements. Phys Med Biol. 51:1759–1768. 16552102 10.1088/0031-9155/51/7/008

[bhw340C96] Thatcher RW, Krause PJ, Hrybyk M 1986 Cortico-cortical associations and EEG coherence: a two-compartmental model. Electroencephalogr Clin Neurophysiol. 64:123–143. 2424729 10.1016/0013-4694(86)90107-0

[bhw340C97] Thevathasan W, Pogosyan A, Hyam JA, Jenkinson N, Bogdanovic M, Coyne TJ, Silburn PA, Aziz TZ, Brown P 2011 A block to pre-prepared movement in gait freezing, relieved by pedunculopontine nucleus stimulation. Brain. 134:2085–2095. 21705424 10.1093/brain/awr131 PMC3122373

[bhw340C98] Thevathasan W, Pogosyan A, Hyam JA, Jenkinson N, Foltynie T, Limousin P, Bogdanovic M, Zrinzo L, Green AL, Aziz TZ, et al 2012 Alpha oscillations in the pedunculopontine nucleus correlate with gait performance in parkinsonism. Brain. 135:148–160. 22232591 10.1093/brain/awr315 PMC3267984

[bhw340C99] Thomson DJ 1982 Spectrum estimation and harmonic analysis. Proc IEEE. 70:1055–1096.

[bhw340C100] Tsang EW, Hamani C, Moro E, Mazzella F, Poon YY, Lozano AM, Chen R 2010 Involvement of the human pedunculopontine nucleus region in voluntary movements. Neurology. 75:950–959. 20702790 10.1212/WNL.0b013e3181f25b35 PMC2942031

[bhw340C101] Valencia M, Chavez M, Artieda J, Bolam JP, Mena-Segovia J 2014 Abnormal functional connectivity between motor cortex and pedunculopontine nucleus following chronic dopamine depletion. J Neurophysiol. 111:434–440. 24174651 10.1152/jn.00555.2013 PMC3921386

[bhw340C102] Van Veen BD, Van Drongelen W, Yuchtman M, Suzuki A, VanVeen BD, vanDrongelen W 1997 Localization of brain electrical activity via linearly constrained minimum variance spatial filtering. Biomed Eng IEEE Trans. 44:867–880. 10.1109/10.623056 9282479

[bhw340C103] Vogt BA 2005 Pain and emotion interactions in subregions of the cingulate gyrus. Nat Rev Neurosci. 6:533–544. 15995724 10.1038/nrn1704 PMC2659949

[bhw340C104] Vrba J, Taulu S, Nenonen J, Ahonen A 2010 Signal space separation beamformer. Brain Topogr. 23:128–133. 19943101 10.1007/s10548-009-0120-7 PMC2874054

[bhw340C105] Wang H-L, Morales M 2009 Pedunculopontine and laterodorsal tegmental nuclei contain distinct populations of cholinergic, glutamatergic and GABAergic neurons in the rat. Eur J Neurosci. 29:340–358. 19200238 10.1111/j.1460-9568.2008.06576.x PMC3833361

[bhw340C106] Winn P 2006 How best to consider the structure and function of the pedunculopontine tegmental nucleus: evidence from animal studies. J Neurol Sci. 248:234–250. 16765383 10.1016/j.jns.2006.05.036

[bhw340C107] Yarnall A, Rochester L, Burn DJ 2011 The interplay of cholinergic function, attention, and falls in Parkinson's disease. Mov Disord. 26:1–8. 10.1002/mds.23932 21898597

[bhw340C108] Yogev-Seligmann G, Hausdorff JM, Giladi N 2008 The role of executive function and attention in gait. Mov Disord. 23:329–342; quiz 472. 18058946 10.1002/mds.21720 PMC2535903

[bhw340C109] Zrinzo L, Zrinzo LV, Tisch S, Limousin PD, Yousry TA, Afshar F, Hariz MI 2008 Stereotactic localization of the human pedunculopontine nucleus: atlas-based coordinates and validation of a magnetic resonance imaging protocol for direct localization. Brain. 131:1588–1598. 18467343 10.1093/brain/awn075

