# Supplementary figures and images for: Functional Connectivity of the Pedunculopontine Nucleus and Surrounding Region in Parkinson's Disease
Source: Cereb Cortex. 2016 Nov 22;27(1):54–67. doi: 10.1093/cercor/bhw340 (PMC5357066; doi:10.1093/cercor/bhw340)

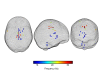

Supplement: Supplementary Data [file Figure_S1.gif]
