# Supplementary material for: Functional Connectivity of the Pedunculopontine Nucleus and Surrounding Region in Parkinson's Disease
Source: Cereb Cortex. 2016 Nov 22;27(1):54–67. doi: 10.1093/cercor/bhw340 (PMC5357066; doi:10.1093/cercor/bhw340)
Supplement: Supplementary Data [file Supplementary_Material3.docx]

**Supplementary Material**

**FUNCTIONAL CONNECTIVITY OF THE PEDUNCULOPONTINE NUCLEUS REGION IN PARKINSON’S DISEASE**

**AUTHORS:** Ashwani Jha*^1,2,3^, Vladimir Litvak*^1,3^, Samu Taulu^4,5^, Wesley Thevathasan^2^, Jonathan A. Hyam^7^, Tom Foltynie^1,7^, Patricia Limousin^1,7^, Marko Bogdanovic^2^, Ludvic Zrinzo^1,7^, Alexander L. Green^6^, Tipu Z. Aziz^6^, Karl Friston^3^ & Peter Brown^2,8^

Supplementary Methods

**Artefact Detection**

Artefacts were detected and marked in continuous data based on the following criteria. Flat segments were defined as 10 or more consecutive samples where the absolute differences between adjacent points were below 1e-10 fT for magnetometers (Oxford) and axial gradiometers (London) or 0.1 fT/mm for planar gradiometers (Oxford). Jumps were defined as changes in amplitude between two consecutive samples of above 50 pT for magnetometers (Oxford), 20 pT for axial gradiometers (London) and 5 pT/mm for planar gradiometers (Oxford). These parameters were defined based on reviewing the raw data and difference time series and looking at the typical values for artefact-contaminated vs. artefact free data segments. A window of -1 to 1 s relative to each jump was also marked as bad. The artefact markings were channel-specific.

**Spatiotemporal Signal Space Separation**

We used Matlab implementation of tSSS provided to us under license by Elekta. This was the prototype code for the commercial implementation available in the MaxFilter™ software. The code was modified to work with data in SPM format and exclude bad data on segment-by-segment basis as will be explained below. The first step in tSSS is projection of the channel data on a multipole basis set. This computation is performed by multiplying the channel data by a complex matrix computed based on the head location with respect to the sensor array. Critically, only a subset of channels can be used as input for the procedure and the missing channels’ activity can be reconstructed by multiplying the multipole complex time series by an inverse projection matrix computed for the complete sensor set and taking the real part. The resulting channel data will be rank-deficient but this is true also with no bad channels because the dimensionality of the multipole set (in our case – 80) is much lower than the number of channels (306). The multipole set determines the physically essential rank, which may lead to numerical problems if the subsequent sensor-level rank is expected to equal the number of channels. Unlike commercial MaxFilter™ software which only excludes bad channels for the complete recording block, our code divided the continuous data into non-overlapping 1 s windows and re-computed the projection matrix for each of these windows using the channels for which no artefacts were marked in the window by the artefact detection routine. This made it possible to still use channels where the signals were valid for substantial part (above 20%) of the recording block while excluding the invalid segments. The resulting multipole time courses were subjected to the second step of tSSS - artefact removal in the temporal domain (correlation limit of 0.98), projected back to the complete sensor set and saved in the output file. We also retained the projection matrix allowing us to re-compute the multipole time-courses from the clean sensor data. It was used for Signal-Space-Separation Beamforming (SSS-BF) analysis as will be described below. In the post-tSSS files the bad channel flags set by artefact detection were removed because the data in all channels appeared artefact-free.

Within-subject source estimation

To investigate, at the individual subject level, how spatial locations of peak coherence vary, especially with regard to the spatial locations identified by the group analysis we generated individual DICS images (as outlined in Methods sections 2.4 and 2.5) based on the individually significant frequency ranges of PPNR-LFP coherence as determined by sensor-level analysis. Here, given the heterogeneous nature of the data, we cannot assume that the global DICS peak in one subject represents a similar source to that in another subject, so any comparison of global peak coherence location is difficult to interpret. To address this, we located the nearest DICS image peak to the canonical locations from the group analysis. To avoid locating peaks that represent local variations in noise, we thresholded the DICS images to only include larger values of coherence (arbitrarily greater than 2 standard deviations above the mean coherence value of the image), which are more likely to represent genuine sources. To partially account for varying signal-to-noise (meaning that some subjects had more individually significant frequency ranges and locations than others) and for the potential repetition of identified locations within a participant (PPNR-LFP coherence with the same cortical location identified in more than one bipolar contact), we only present the one nearest cortical source to each canonical brain location per side per patient.
